# Season affects the estrogen system and the immune response of common carp

**DOI:** 10.1007/s10695-023-01286-2

**Published:** 2023-12-29

**Authors:** Magdalena Maciuszek, Lukasz Pijanowski, Lidy Verburg-van Kemenade, Magdalena Chadzinska

**Affiliations:** 1 Department of Evolutionary Immunology, Institute of Zoology and Biomedical Research, Faculty of Biology, Jagiellonian University, Krakow, Poland; 2 Cell Biology and Immunology Group, Department of Animal Sciences, Wageningen University, Wageningen, the Netherlands

**Keywords:** Common carp, Seasonality, Immune response, Estrogen system, Head kidney, Liver

## Abstract

The physiology of ectothermic animals, including fish, is strictly regulated by season-related external factors such as temperature or photoperiod. The immune response and the production of hormones, such as estrogens, are therefore also subject to seasonal changes. This study in common carp aimed to determine how the season affects the estrogen system and the immune response, including the antibacterial response during *Aeromonas salmonicida* infection. We compared the immune reaction in spring and autumn in the head kidney and liver and found that carp have higher levels of blood 17β-estradiol in autumn, while in the liver of these fish there is a higher constitutive expression of genes encoding vitellogenin, estrogen receptors and Cyp19 aromatase than in spring. Fish sampled in autumn also exhibited higher expression of immune-related genes in the liver. In contrast, in the head kidney from fish sampled in the autumn, the expression of genes encoding estrogen receptors and aromatase was lower than in spring, and a similar profile of expression was also measured in the head kidney for *inos*, *arginases* and *il-10*. In turn, during bacterial infection, we observed higher upregulation of the expression of *inos*, *il-12p35*, *ifnγ-2*, *arginase 2* and *il-10* in the liver of carp sampled in spring. In the liver of carp infected in spring a higher upregulation of the expression of the genes encoding CRPs was observed compared to fish infected during autumn. The opposite trend occurred in the head kidney, where the upregulation of the expression of the genes involved in the immune response was higher in fish infected in autumn than in those infected in spring. During the infection, also season-dependent changes occurred in the estrogen system. In conclusion, we demonstrated that season differentially affects the estrogenic and immune activity of the head kidney and liver. These results reinforce our previous findings that the endocrine and immune systems cooperate in maintaining homeostasis and fighting infection.

**Supplementary Information:**

The online version contains supplementary material available at 10.1007/s10695-023-01286-2.

## Introduction

The immune response of fish is significantly influenced by various external factors, including temperature and light (Magnadottir 2010). These factors drastically differ between seasons and cause season-dependent changes in the immunological parameters such as hematocrit, the number of leukocytes and their activity (respiratory burst, phagocytosis and nitrite production (Saha et al. 2002; Buchtíková et al. 2011; Montero et al. 2022; Zheng et al. 2022). Moreover, the production of antibacterial proteins and IgM, as well as the activity of the complement system, is often reported to be season dependent (Saha et al. 2002; Buchtíková et al. 2011; Dolan et al. 2016; Kondera et al. 2019; Bhardwaj et al. 2022). Furthermore, exposure to many parasites and pathogens varies seasonally, following the parasite life cycle or because of intra-annual variation in the infectivity of pathogens (Banerjee and Bandyopadhyay 2010; Rohlenová et al. 2011; Szwejser et al. 2017b).

Interactions between the endocrine and immune systems enable the body to maintain homeostasis and to adapt to changing internal and external environmental factors. They are based on a common language of signaling molecules and receptors (Verburg-van Kemenade et al. 2017). However, these relationships are quite complex, and this complexity increases even more, due to the fact that the activity of the endocrine system, including the estrogen system, is also affected by external factors such as ambient temperature, light, water quality, oxygen level and salinity (Hjernquist et al. 2012; Szwejser et al. 2017b; Gotthard 2001). The seasonal nature of hormone concentration levels in fish is well-known. For example, in common carp higher concentrations of 17β-estradiol (E2) were usually observed in April and November, while their lowest concentrations occur in August (Saha et al. 2002; Taghizadeh et al. 2013). In turn, in rainbow trout, a higher level of E2 was reported in May and in September (Chen et al. 2021). In addition, high E2 concentrations affect the level of vitellogenin, as well as the expression of estrogen receptors and aromatase (Nelson et al. 2007; Nagler et al. 2012; Ye et al. 2022).

It must be underlined that in fish, like in other vertebrates, steroid hormones, including estrogens, are important regulators of the immune system (Segner et al. 2017; Verburg-van Kemenade et al. 2017). Fish leukocytes express nuclear (ERα, ERβ) and membrane (GPER1/GPR30) estrogen receptors (Iwanowicz et al. 2014; Kovats 2015; Szwejser et al. 2017a), and the most common cellular effect of estrogen-induced immunoregulation is the inhibition of NFκB activity and the regulation of MAPK kinase activity (Biswas et al. 2005). These pathways decrease the expression of proinflammatory mediators (Ghisletti et al. 2005; Burgos-Aceves et al. 2016), reduce the production of reactive oxygen species (ROS) and nitric oxide (NO) (Shelley et al. 2013; Iwanowicz et al. 2014), phagocytosis (Seemann et al. 2015) and lysozyme activity (Iwanowicz et al. 2014). Moreover, E2 affects the adaptive immune response, but in this case, results obtained in different fish species are conflicting (Suzuki et al. 1996; Cuesta et al. 2007; Thilagam et al. 2009). For example, E2 increased B-cell proliferation and antibody production in rainbow trout and crucian carp (Suzuki et al. 1996; Thilagam et al. 2009; Cook 1994), while it decreased B-cell activity in sea bream (Cuesta et al. 2007). Moreover, E2 exposure induced thymic involution and thymocyte differentiation (Seemann et al. 2015). E2 also decreased mitogen-stimulated proliferation of blood leukocytes of channel catfish and rainbow trout (Shelley et al. 2013; Iwanowicz et al. 2014).

Both in mammals and in fish, estrogen-induced immunomodulation is strictly dependent on the hormone concentration and the exposure time (Cabas et al. 2013). For example, Cabas and co-workers (Cabas et al. 2013) showed a decrease of phagocytosis in seabream head kidney cells treated *in vitro* with E2 for 1 h, while the opposite phenomenon was found in cells incubated with E2 for 16 and 48 h. Moreover, high concentrations of estradiol (10–1000 nM) reduced phagocytosis in the head kidney leukocytes of carp (Yamaguchi et al. 2001), while a lower concentration (5.5 nM) decreased the ROS production in the head kidney cells of seabream (Chaves-Pozo et al. 2003). In turn, in goldfish, high E2 dose (5 μM) suppressed the *in vitro* chemotaxis of cells from a kidney macrophage cell line (Wang and Belosevic 1995), while 1 μM of E2 downregulated both the expression of pro- and anti-inflammatory mediators in carp monocytes/macrophages (Maciuszek et al. 2020a). However, macrophages of seabream that were stimulated with *Vibrio anguillarum* genomic DNA (*Va*DNA) increased their expression of pro-inflammatory mediators upon E2 treatment (18.35 or 183.55 nM) (Liarte et al. 2011).

Furthermore, *in vivo* studies in fish demonstrated that estradiol influenced the outcome of disease. It significantly reduced the mortality of zebrafish infected with spring viraemia of carp virus (SVCV) (López-Muñoz et al. 2015), while it increased the mortality of goldfish infected with *Trypanosoma danilewsky* (Wang and Belosevic 1994). Also, in rainbow trout infected with *Yersinia ruckeri*, both long (at least 6 months) and short-term (14 days) exposure to E2 resulted in increased mortality (Wenger et al. 2011). Such E2-exposure also decreased the expression of complement components (*c3-1, c3-3* and *factor-H*) in the liver of *Yersinia ruckeri*-infected fish. In turn, our studies found that in carp fed for 14 days with an E2-containnig diet, the expression of anti-inflammatory mediators during *Aeromonas salmonicida* infection was higher than in fish fed with control diet (Maciuszek et al. 2020a).

Interestingly, immune cells not only respond to hormones but are also capable to produce hormones. For example, carp lymphoid organs (thymus, spleen, head kidney) and leukocytes (lymphocytes, monocytes/macrophages and neutrophilic granulocytes) express the *cyp19a* and *cyp19b* encoding aromatase cytochrome P450, involved in the conversion of C19 steroids to estrogens (Szwejser et al. 2017b).

Data about estrogen-induced gender-dependent differences in the immune response in vertebrates are very limited as most studies are performed with male individuals. In medaka infected with *Edwardsiella tarda*, high endogenous E2 levels caused higher mortality of female fish than male fish (Dong et al. 2017). Also in zebrafish, *cyp19a1a* was identified as a putative factor involved in this feature as during SVCV infection, *cyp19a1a* ^*−/−*^ zebrafish males have higher expression of *ifnφ1* and lower expression of the viral *svcv-n* gene than *cyp19a1a*^*+/+*^ males and females. Moreover, it was demonstrated that MITA, a crucial mediator of virus-triggered type I IFN signaling, colocalizes with Cyp19a1a in the endoplasmic reticulum and that Cyp19a1a is a negative regulator of expression of type I IFNs (Lu et al. 2022).

It however remains poorly understood to what extent the estrogen system in fish is responsible for the seasonal changes of the immune response.

The trade-off theory presumes that during the reproductive period, with higher levels of E2, other processes and physiological functions are inhibited to save energy in favor of reproduction (Sueiro and Palacios 2016). However, it must be emphasized that this compromise is not a fixed value and can be optimized according to the actual needs of the organism. Also, costs related to the immune system include costs for recovery and tissue repair after infection or reproduction, e.g., increased protein synthesis (Vargas-Villavicencio et al. 2009).

In the present study, we aim to establish whether the season-related differences in the antimicrobial/inflammatory response of carp are related to the seasonal changes in the estrogen system.

## Materials and methods

### Animals

Young sexually immature individuals of common carp (*Cyprinus carpio* L; 9–12 months; line R3×R8; 60–90 g) were obtained from the Institute of Ichthyobiology and Aquaculture, Polish Academy of Science, Golysz, Poland. Studies were conducted in the autumn (November/December 2018) and in the spring (March/April 2019). Fish were exposed to the natural photoperiod occurring in Poland during mid-autumn (9 h of light and 15 h of dark) and mid-spring (12 h of light and 12 h of dark). The water temperature was constant at 20–21 °C. Prior to the experiments, fish were adapted to their new environment for 4 weeks at 21 °C in recirculating tap water at the Institute of Zoology and Biomedical Research in Krakow, Poland. Fish were kept in tanks (volume 375 l, flow rate 4 l/min, density 45 fish/tank and 60 g/l), and all unnecessary interferences were avoided. Fish were fed pelleted dry food (Aller Master, Aller Aqua, Poland) at a daily maintenance rate of 1% of their estimated body weight. To avoid additional stress and/or differences in handling, all samplings were performed by the same person and at the same time of day (at 9.00 am).

All animals were handled in strict accordance with good animal practice as defined by the relevant national and local animal welfare bodies, and procedures were approved by the local ethical committee (2nd Local Institutional Animal Care and Use Committee (IACUC) in Krakow, Poland, license number 291/2017).

### Infection

In both seasons, fish were kept in 6 tanks (40 l, with water circulation and aeration). Experiments were performed 2 times independently in both seasons, with 3–4 fish per group/time point every time (CTR, 24 hpi, 96 hpi).

*Aeromonas salmonicida* subsp. salmonicida from Polish origin was obtained from the Department of Fish Diseases, National Veterinary Research Institute, Pulawy. Bacteria were grown in lysogeny broth (LB) medium for 18 h at 25 °C, centrifuged at 1600×g for 10 min and the bacterial pellet reconstituted in sterile PBS (280 mOsM) as described previously (Maciuszek et al. 2020a, b). Optical density was measured at 625 nm, and data were aligned with a previously derived McFarland scale to determine the bacterial concentration.

Fish were anesthetized (5 min) with tricaine methane sulphonate (TMS; Sigma-Aldrich, St. Louis, MO, USA; 0.2 g/l) buffered with NaHCO_3_ (POCH, Gliwice, Poland; 0.4 g/l) and intraperitoneally injected with a non-lethal dose of *A. salmonicida* (4 × 10^8^ bacteria in 250 μl PBS per fish) as described previously (Falco et al. 2012). Fish were sacrificed at 24 and 96 h post-injection (hpi, *n*=7 per each group). Control animals were kept untreated. All fish were sacrificed by deep and prolonged anesthesia with TMS.

### Serum hormone level

Fish were bled through puncture of the caudal vein using a needle attached to a 5 ml syringe. The samples were taken midline just posterior of the anal fin. Every time approximately 5 ml of blood was removed from the caudal vein into a syringe. Blood clots were removed by centrifuging at 800×g for 10 min, and serum was collected and stored at −20 °C for future use.

Hormone levels were determined using commercial kits: (i) for estradiol: DRG, Marburg, Germany (range 10.6–2000 pg/ml, sensitivity 10.60 pg/ml, (ii) for cortisol: Neogen Kit, Lexington, USA (range 0.04–10.0 ng/ml; sensitivity 0,04 ng/ml). Analyses were performed according to the manufacturer’s protocol. All standards and samples from every individual fish were analyzed in duplicate, in the same batch.

### Gene expression

Head kidneys and livers of control and infected fish were carefully removed and immediately transferred to fix RNA (EURx, Gdansk, Poland) and kept at −20 °C for further analysis.

RNA was isolated from tissues with GeneMATRIX Universal RNA Purification Kit (EURx, Gdansk, Poland) according to the manufacturer’s protocol. Final elution was carried out in 30 μl of nuclease-free water, to maximize the concentration of RNA. Before proceeding with further analyses, RNA was quantified, and its integrity checked (Tecan Spark NanoQuant PlateTM). Samples were stored at −80 °C.

For each sample, a non-RT (non-reverse transcriptase) control was included. The cDNA synthesis was performed with High-Capacity cDNA Reverse Transcription Kits (Applied Biosystems, Waltham, Massachusetts, USA) according to the manufacturer’s protocol. Briefly, 1 μg of total RNA was added to 10 μl RT master mix containing 2 μl 10X RT Buffer, 0.8 μl 25XdNTP Mix (100 mM); 2 μl 10XRT Random Primers; 1 μl MultiSribe™Reverse Transcriptase and 4.2 μl of nuclease-free water. Samples were then placed into the thermal cycler (Ditabis AG, Pforzheim, Germany, 25 °C at 10 min; 37 °C at 120 min; 85 °C at 5 min followed by rapid cooling to 4 °C). Samples were set at 100 μl with nuclease-free water and stored at −20 °C until further use.

Carp-specific primers (5′–3′) for immune-related (*inos*, *il-1β*, *il-12p35 ifn-γ2*, *c3*, *crp1*, *crp2*, *arginase 1*, *arginase 2*, *il-10*) and endocrine-related (*erα*, *erβ*, *gper1*, *cyp19a*, *cyp19b*, *vtg*) RNA detection were used. The 40S ribosomal protein s11 (*40s11*) gene served as an internal standard. Accession numbers, primer sequences and their concentrations are listed in Supplementary Table 1.

For RT-qPCR 4 μl cDNA and forward and reverse primers (2 μl each) were added to 7 μl SYBR®Select Master Mix (Applied Biosystems, Waltham, Massachusetts, USA). RT-qPCR (2 min at 50 °C, 2 min at 95 °C, 40 cycles of 15 s at 95 °C, 60 s at 60 °C) was carried out with a Rotor-Gene Q (Qiagen, Hilden, Germany). Following each run, melt curves were collected by detecting fluorescence from 60 to 90 °C at 1 °C intervals.

Constitutive expression was rendered as a ratio of target gene vs. reference gene (40S ribosomal protein s11 gene) and was calculated according to the following equation:

$$\textrm{Ratio}=\frac{{\left({E}_{reference}\right)}^{Ct_{reference}}}{{\left({E}_{target}\right)}^{Ct_{target}}}$$

where E is the amplification efficiency and Ct is the number of PCR cycles needed for the signal to exceed a predetermined threshold value (Pfaffl 2001).

Changes in gene expression upon seasons, between autumn and spring, were rendered as a ratio of target gene vs. reference gene (40S ribosomal protein s11 gene) relative to expression in control samples according to the following equation:

$$\textrm{Ratio}=\frac{{\left({E}_{target}\right)}^{\Delta Ct}{Target}^{\left( control- sample\right)}}{{\left({E}_{reference}\right)}^{\Delta Ct}{Reference}^{\left( control- sample\right)}}$$

### Statistical analysis

Analysis mRNA levels were performed in Microsoft Excel, and statistical analysis was performed with GraphPad 8 Software (San Diego, CA, USA). Data were expressed as mean and standard error (SE). The assumptions of normality and equality of variance were found. Shapiro-Wilk and Gaussian distribution for normality test, and F test and Brown-Forsythe test for variances, were used. Differences in the serum level of E2 and constitutive gene expression between autumn and spring were compared using the Mann-Whitney *U* test. Significance of differences in *in vivo* study were compared by two-way analysis of variance (ANOVA), followed by post hoc Tukey’s test for multiple comparisons. Results of interactions in two way ANOVA analysis are included in Supplementary Table 2. The differences were considered statistically significant at *p*<0.05.

## Results

### Season-dependent differences in the estrogen system

**Fig. 1:**
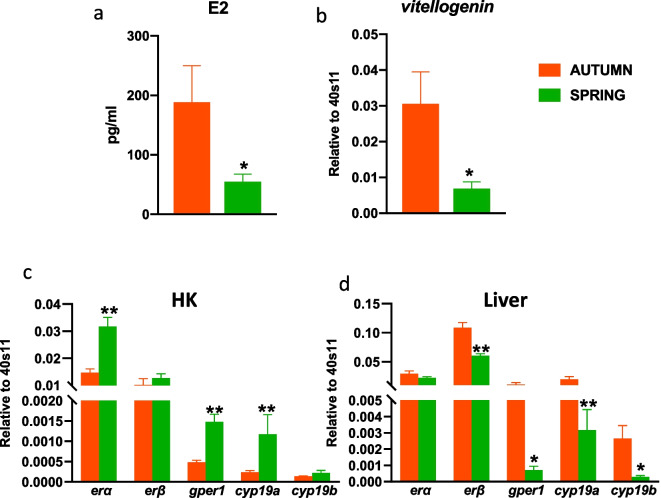
Season-dependent differences in the estrogen system. **a**—level of E2 in blood plasma, **b**—gene expression of vitellogenin in the liver, **c**, **d**—gene expression of estrogen receptors (*erα, erβ* and *gper1*) and enzymes involved in E2 conversion (*cyp19a* and *b*) in the head kidney (HK) and liver. Constitutive gene expression was determined by quantitative RT-qPCR and expressed relative to the expression of the 40S ribosomal protein s11 gene. Averages and SE (*n*=5–7). Stars indicates significant differences between autumn (orange bar) and spring (green bar) (* *p* ≤ 0.05, ** *p* ≤ 0.01)

In autumn, the level of plasma 17β-estradiol and the gene expression of vitellogenin (*vtg*) in the liver were significantly higher than in the spring (Fig. 1a, b). The constitutive expression of genes encoding estrogen receptors (ERα, GPER1) and *cyp19a* in the head kidney was significantly lower in autumn compared to spring (Fig. 1c). In contrast, in the liver, constitutive expression of genes encoding estrogen receptors (ERβ and GPER1), as well as enzymes involved in the E2 conversion (CYP19a and b) was higher in the autumn than in the spring (Fig. 1d).

### Season-dependent differences in the constitutive gene expression of inflammatory markers

**Fig. 2:**
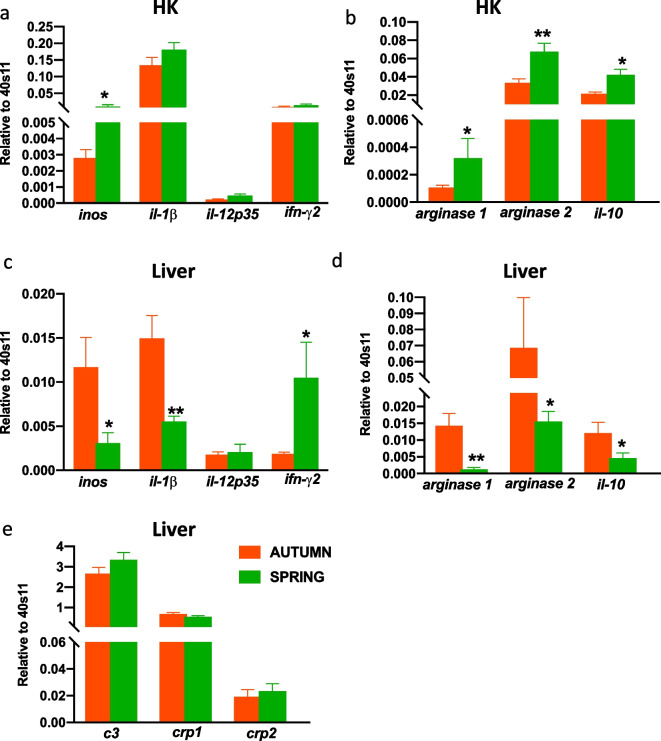
Season-dependent differences in the constitutive expression of inflammatory mediators in the head kidney (**a**, **b**—HK) and liver (**c**—**e**). Constitutive gene expression of proinflammatory (*inos*, *il-1β*, *il-12p35*, *ifn-γ2*), anti-inflammatory (*arginase 1*, *arginase 2*, *il-10*) mediators and acute phase proteins (*c3*, *crp1*, *crp2*) was determined by quantitative RT-qPCR and expressed relative to the expression of the 40S ribosomal protein s11 gene. Averages and SE (*n*=5–7). Stars indicates significant differences between autumn (orange bar) and spring (green bar) (* *p* ≤ 0.05, ** *p* ≤ 0.01)

Lower constitutive expression *of inos*, *arginase 1* and *2*, *il-10* was observed in the head kidney in the autumn (Fig. 2a, b). In the liver, only *ifn-γ2* expression was lower during this season (Fig. 2c). In the liver constitutive expression of *inos*, *il-1β*, *arginase 1*, *arginase 2* and *il-10* was lower in the spring than in the autumn (Fig. 2c, d). Constitutive expression of genes encoding C3, CRP1 and CRP2 did not differ between both seasons (Fig. 2e).

### Season-dependent and infection-induced differences in gene expression of inflammatory markers during bacterial infection

**Fig. 3:**
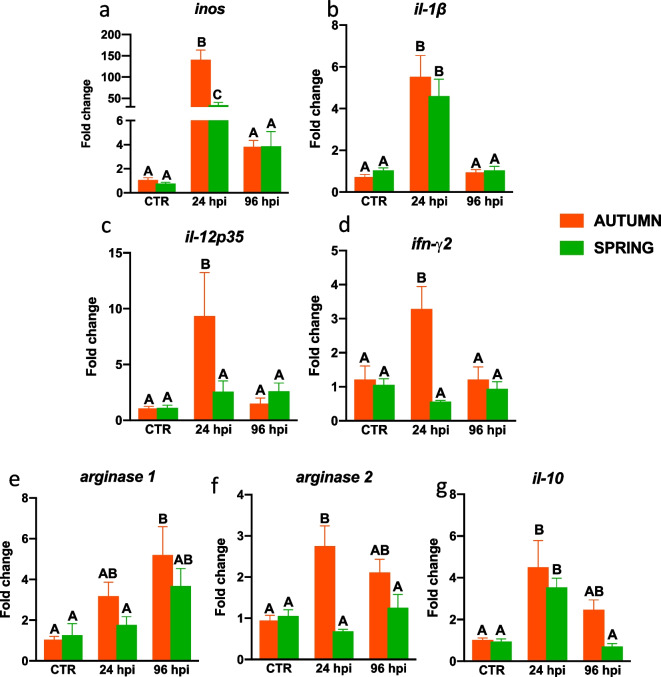
Season-dependent differences in the gene expression of inflammatory markers in the head kidney of *Aeromonas salmonicida* infected fish. Fish were i.p. injected with *A. salmonicida* (4 × 10^8^ bacteria in 250 μl PBS per fish). At 24 and 96 h post-infection (hpi) the head kidneys were collected, and gene expression of proinflammatory (**a**—*inos*, **b**—*il-1β*, **c**—*il-12p35*, **d**—*ifn-γ2*) and anti-inflammatory (**e**—*arginase 1*, **f**—*arginase 2*, **g**—*il-10*) mediators was measured by quantitative RT-qPCR. Averages and SE (*n*=5–7). Changes in gene expression are shown as x-fold increase compared to the control group without infection (CTR) and standardized for the housekeeping gene 40S ribosomal protein s11. Mean values not sharing letters are statistically different between autumn (orange bar) and spring (green bar)

**Fig. 4:**
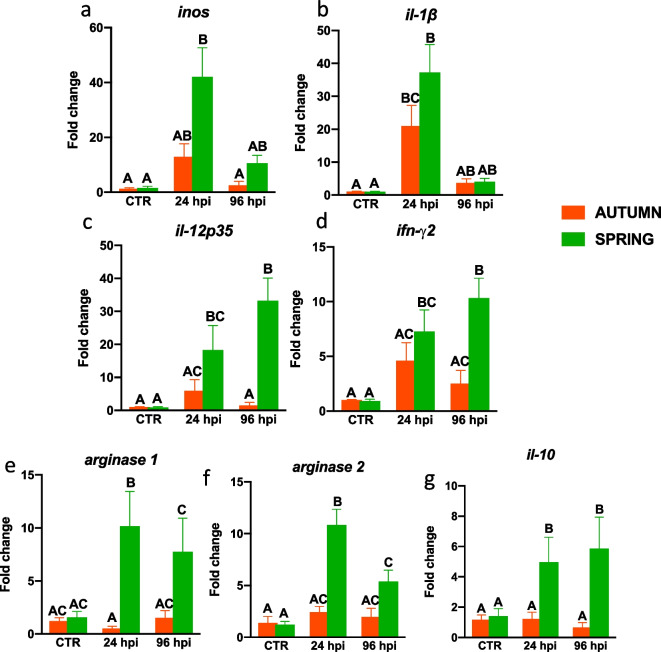
Season-dependent differences in the gene expression of inflammatory markers in the liver in *Aeromonas salmonicida* infected fish. Fish were i.p. injected with *A. salmonicida* (4 × 10^8^ bacteria in 250 μl PBS per fish). At 24 and 96 h post-infection (hpi) the livers were collected, and gene expression of proinflammatory (**a**—*inos*, **b**—*il-1β*, **c**—*il-12p35*, **d**—*ifn-γ2*), anti-inflammatory (**e**—*arginase 1*, **f**—*arginase 2*, **g**—*il-10*) mediators was measured by quantitative RT-qPCR. Averages and SE (*n*=5-7). Changes in gene expression are shown as x-fold increase compared to the control group without infection (CTR) and standardized for the housekeeping gene 40S ribosomal protein s11. Mean values not sharing letters are statistically different between autumn (orange bar) and spring (green bar)

In both seasons, at 24 hpi, the expression of genes encoding for IL-1β and IL-10 was upregulated in the head kidney (Fig. 3a, b, g). Upregulation of *inos* expression was significantly higher in autumn than in spring. Upregulation of the expression of *il-12p35, ifn-γ2* and *arginase 2* in the head kidney was only found in the autumn (Fig. 3c, d, f). In contrast, in the liver, the infection-induced upregulation of the expression of inflammatory markers was mainly observed in the spring. In spring, infection induced upregulation of *inos* at 24 hpi (Fig. 4a), while upregulated expression of *il-12p35, ifn-γ2*, *arginase 2* and *il-10* was observed only in the spring both at 24 and 96 hpi (Fig. 4c–g). Upregulation of *il-1β* expression was also found in the liver in the autumn at 24 hpi (Fig. 4b).

**Fig. 5:**
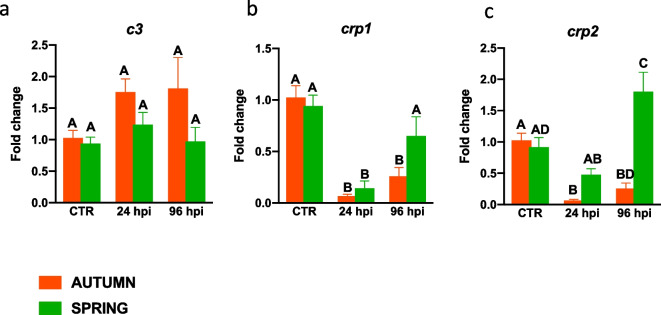
Season-dependent differences in the gene expression of acute phase proteins in the liver in *Aeromonas salmonicida* infected fish. Fish were i.p. injected with *A. salmonicida* (4 × 10^8^ bacteria in 250 μl PBS per fish). At 24 and 96 h post-infection (hpi) the livers were collected, and gene expression of acute phase proteins (**a**—*c3*, **b**—*crp1*, **c**—*crp2*) was measured by quantitative RT-qPCR. Averages and SE (*n*=5–7). Changes in gene expression are shown as x-fold increase compared to the control group without infection (CTR) and standardized for the housekeeping gene 40S ribosomal protein s11. Mean values not sharing letters are statistically different between autumn (orange bar) and spring (green bar)

In the liver, infection downregulated the expression of *crp1* (at 24 hpi in the autumn and spring and at 96 hpi only in the autumn) and *crp2* (at 24 and 96 hpi in the autumn) (Fig. 5), while in the spring, at 96 hpi, expression of *crp2* was upregulated (Fig. 5c).

### Season-dependent and infection-induced changes in the expression of receptors and enzymes from the estrogen system

**Fig. 6:**
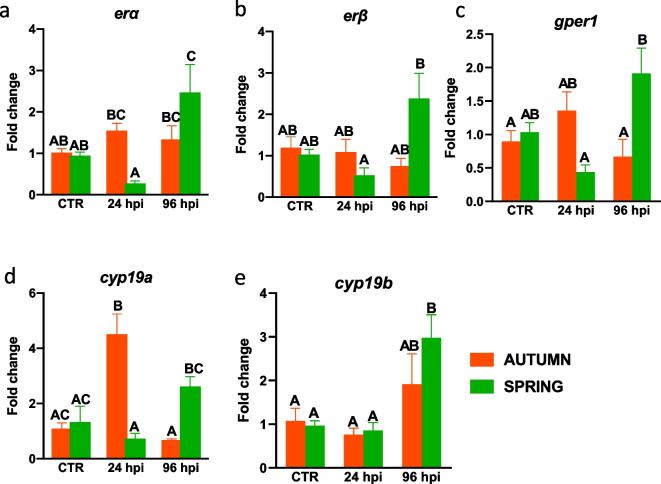
Season-dependent and infection-induced changes in estrogen system in the head kidney. Fish were i.p. injected with *A. salmonicida* (4 × 10^8^ bacteria in 250 μl PBS per fish). At 24 and 96 h post-infection (hpi) the head kidneys were collected, and gene expression of estrogen receptors (**a**—*erα*, **b**—*erβ* and **c**—*gper1*) and enzymes involved in E2 conversion (**d**—**e** *cyp19a* and *b*) was measured by quantitative RT-qPCR. Averages and SE (*n*=5–7). Changes in gene expression are shown as x-fold increase compared to the control group without infection (CTR) and standardized for the housekeeping gene 40S ribosomal protein s11. Mean values not sharing letters are statistically different between autumn (orange bar) and spring (green bar)

In the spring, expression of *erα*, *gper1* and *cyp19b* was upregulated in the head kidney at 96 hpi compared to the expression observed in control animals (Fig. 6a, c, e). A similar phenomenon was not observed in the autumn. In this season only infection-induced upregulation of *cyp19a* expression was found at 24 hpi (Fig. 6d).

**Fig. 7:**
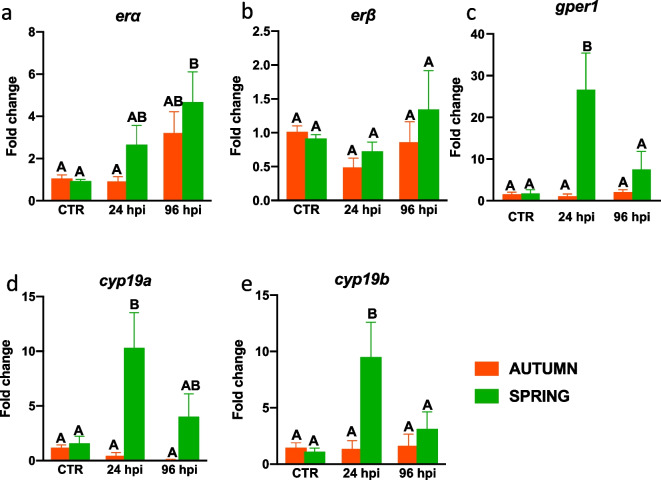
Season-dependent and infection-induced changes in the expression of estrogen system genes in the liver. Fish were i.p. injected with *A. salmonicida* (4 × 10^8^ bacteria in 250 μl PBS per fish). At 24 and 96 h post-infection (hpi) the livers were collected, and gene expression of estrogen receptors (**a**—*erα*, **b**—*erβ* and **c**—*gper1*) and enzymes involved in E2 conversion (**d**—**e**, *cyp19a* and *b*) was measured by quantitative RT-qPCR. Averages and SE (*n*=5–7). Changes in gene expression are shown as x-fold increase compared to the control group without infection (CTR) and standardized for the housekeeping gene 40S ribosomal protein s11. Mean values not sharing letters are statistically different between autumn (orange bar) and spring (green bar)

In the liver, during spring, *A. salmonicida* infection induced upregulation of *gper1*, *cyp19a* and *cyp19b* (at 24 hpi) (Fig. 7c–e) and *erα* (at 96 hpi) (Fig. 7a).

The level of 17β-estradiol in the blood plasma did not differ between control and infected animals in the spring, while in the autumn it was lower in infected fish (at 24 hpi) than in control uninfected animals and in fish at 96 hpi (Fig. S1). The levels of cortisol in the blood plasma did not differ in all examined samples (Fig. S2).

## Discussion

We identified seasonal differences in the antimicrobial/inflammatory response of common carp and observed distinctive differences between the estrogen system in the head kidney and liver.

When comparing the activity of the estrogen system of carp throughout spring and autumn we found that fish sampled in the autumn exhibited higher levels of plasma E2. Previously such differences, both in the level of blood and gonadal E2, were found in several fish species (Saha et al. 2002; Taghizadeh et al. 2013; Soranganba 2022).

In the present studies, in the liver of fish sampled during springtime, we also observed higher constitutive expression of genes encoding vitellogenin, a precursor of egg yolk, estrogen receptors (ERα, GPER1) and estrogen synthase (CYP19), responsible for the biosynthesis of estrogens. Like in mammals, fish liver plays a crucial role in digestion, in energy metabolism, xenobiotic detoxification, biosynthesis of serum proteins and also in endocrine and immune responses (Taylor et al. 2022). The liver also participates in steroidogenesis (Rajakumar and Senthilkumaran 2020). In oviparous animals, such as teleost fish, the female liver is the main organ for the synthesis of oocyte constituents, such as vitellogenins and the zona pellucida proteins (choriogenins and other minor vitamin-binding proteins). Their synthesis is under direct control of estrogens (Qiao et al. 2016). In many fish species, the liver undergoes seasonal variations in size and in the content of fat and glycogen, and these changes depend on the fish gender and estrogen concentrations, as they can be abolished either by ovariectomy in females or by the administration of estrogen to mature males (Ishii and Yamamoto 1970). Recently, Chen and coworkers (Chen et al. 2021) described season-related differences in the gene expression of estrogen receptors and vitellogenin in the liver of rainbow trout. Similar to our observation for carp, the constitutive expression of these genes in rainbow trout was higher in the course of autumn (October–November) than in the spring. Also, compared to the breeding females (sampled in April), non-breeding females of the orange-spotted grouper (sampled in February) had lower expression of *vtg*, *erα*, *erβ1* and *erβ2* in the liver (Ye et al. 2022).

Interestingly, in the liver of carp we now also observed season-related differences in the expression of the genes involved in the immune response. During autumn, the constitutive expression of genes encoding iNOS, IL-1β, arginase 1 and 2 as well as IL-10 was higher than in the spring. We did not observe such differences in the constitutive expression of genes encoding for acute phase proteins. In contrast, a recent study of Zheng and coworkers (Zheng et al. 2022) in females of Chinese sturgeons showed lower levels of plasma CRP in autumn and winter than in spring and summer, whereas in males lower CRP values were registered throughout the first three seasons and they increased in winter. Previously, seasonal differences in the expression of genes encoding immune mediators were also observed in the liver of Nile tilapia, for which higher expression of these genes was measured in summer and late summer. However, for this experiment, the fish were sampled in their natural living environment, and therefore the authors concluded that these differences correlated with variations during different seasons in the water temperature, oxygen level, concentrations of ammonia and nitrite, as well as with water contamination (Haredi et al. 2020).

We also verified if the antibacterial immune response in carp differs between autumn and spring. Fish were infected with *Aeromonas salmonicida* which causes in carp erythrodermatitis manifested by hemorrhagic changes and inflammation (Falco et al. 2012; Pionnier et al. 2013; Maciuszek et al. 2020a, b). In common carp, *A. salmonicida* do not cause sepsis, although due to the influence of secondary infections and their consequences, it can cause fish death (mortality less than 20%) (Molnár et al. 2019).

In the liver of *A. salmonicida*-infected fish we found upregulation of the gene expression of several pro- and anti-inflammatory mediators, and surprisingly these increases were more pronounced in fish sampled in the spring compared to those sampled in the autumn. Previously, *A. salmonicida*-induced changes in the expression of some immune-related genes were observed by Pionnier and colleagues (Pionnier et al. 2013); however, the authors did not specify the season in which these experiments were performed. Similarly, Dietrich and co-workers (Dietrich et al. 2022) found that at 48 h of *A. salmonicida* infection, the expression of *tnfα*, *il-6a*, but not *ifng-2a*, is upregulated in the liver of carp. In turn, in turbot, an infection with *A. salmonicida* induced upregulation of immune related genes such as MHCI and II, IgGFcR1, C1qB and nitric oxide synthase trafficker, and downregulation of genes associated with the acute phase response and metabolic processes (Millán et al. 2011).

Moreover, in spring, bacterial infection also caused an upregulation of the expression of genes encoding for membrane estrogen receptor GPER1 and CYP19 (24 hpi) in the liver.

Throughout these seasons, we also measured the activity of the estrogen and immune system in the head kidney, which in teleost fish comprises a lymphoid organ, a major hematopoietic organ and an endocrine gland producing cortisol, catecholamines and thyroid hormones (Geven and Klaren 2017; Verburg-van Kemenade et al. 2017). In contrast to the results obtained for the liver, in the head kidney of fish that were sampled in the autumn, we found lower constitutive expression of the genes encoding estrogen receptors (ERα and GPER1) and CYP19a than in spring. Also, the constitutive expression of immune-related genes in the head kidney, such as *inos*, *arginase 1* and *2* and *il-10*, was lower in autumn than in the spring.

In turn, the results in three-spined sticklebacks showed that during spring there is an increased expression of genes involved in the adaptive immune response (*cd8a*, *foxp3b*, *orai1*, *il-1r-like*, *tbk1*), while late winter was characterized by signatures of innate immunity (including IL-1 signaling and non-classical complement activity) and adjusted toll-like receptor signaling (Brown et al. 2016). Moreover, in goldfish, differences in the expression of genes related to the innate immune response were observed both between the breeding (November) and non-breeding season (March), but also between males and females (Zhong et al. 2014). During the breeding season they found an increased expression of *tnfα1* and *2*, *ifn-γ*, *ccl-1* and *cxcl8* in the head kidney of females, compared to females in non-breeding season. Moreover, in the head kidney of males they did not observe such changes. Interestingly, in the other organs (spleen, gill and liver) higher expression of these genes was noticed in females sampled during the breeding season, compared to non-breeding females and males.

Like in the liver, also in the head kidney *A. salmonicida* infection up-regulated expression of genes involved in the inflammatory reaction; however, in this case strong upregulations were observed in the head kidney of fish sampled in the autumn. Previous studies of carp found that after intraperitoneal injection with *A. salmonicida*, at 6 hpi there was an increase in the expression of genes encoding pro-inflammatory mediators (*tnfα1* and *2*) and anti-inflammatory *il-10*. Interestingly, no changes were observed at the later time points studied (12 h – 5 days) (Falco et al. 2012). Moreover, for *A. salmonicida*-infected crucian carp, Ling and coworkers (Ling et al. 2019) observed elevated expression of *il-1β*, *tnfα*, *il-11* and *c-lysozyme*. Moreover, elevated expression of pro-inflammatory genes was also demonstrated in the head kidney of infected carp by the Pionnier and colleagues (Pionnier et al. 2013). They found increased expression of *crp1*, *bf/c2, c3* and *masp2*. In, turn, in turbot, after infection with *A. salmonicida* an upregulation of genes related to the immune/defense response was observed (Millán et al. 2011). Unfortunately, these publications did not study the impact of season. In turn, Buchtíková et al. (2011) have shown in carp blood plasma higher activity of the alternative complement pathway in November and April than in other months, what may suggest its important protective role before wintertime and spawning period.

In wintertime, Montero and coworkers (Montero et al. 2022) found a decreased number of lymphoid cells in the head kidney, spleen and thymus of rainbow trout that were kept under constant photoperiod and water temperature throughout the year. In carp, changes in the number of T- and B-cells and their activity were observed in spring (May), summer (August) and autumn (October). An increase in the number of T-cells and a decrease in number of B-cells were demonstrated in summer and autumn (Rudenko et al. 2019).

Moreover, upon *A. salmonicida* infection of rainbow trout, seasonal differences in the immune response, with decreased B- and T-cell responses in the winter, were described. Also, a strong increase in the level of natural, but not specific, antibody levels only appeared in fish (at 12 hpi) during the wintertime. Similar results indicating a decrease of leukocyte numbers and immunosuppression in response to the antigen during winter were published for other fish species including carp (Wishkovsky and Avtalion 1987; Saha et al. 2002; Rijkers and Frederix-Wolters 1980). For example, in rainbow trout it was found that phagocytosis, complement lytic activity, respiratory burst activity and opsonization capacity of plasma were higher at 20 °C than at 5–10 °C (Nikoskelainen 2004).

In overwintering channel catfish that were challenged with *Aeromonas hydrophila*, Yang and coworkers (Yang et al. 2015) observed higher expression of *il-1β* and *il-8* in the head kidney between December and April.

We observed a convincing seasonal correlation between the constitutive expression of genes that encode for the estrogen system and the inflammatory mediators both in the liver (higher expression of both groups of genes in the autumn than in the spring) and in the head kidney (higher expression of both groups of genes in the spring than in the autumn). We however do not have direct proof that E2, via estrogen receptors, differentially regulates the immune response in both organs in spring- and autumn-time. Nevertheless, our previous studies confirmed that, during spring time, E2-supplementation changes the expression of immune-related genes in the head kidney and liver of *A. salmonicida*-infected carp (Maciuszek et al. 2020a). Results describing immunomodulatory effects of E2 were also published for rainbow trout during *Yersenia ruckeri* infection (Wenger et al. 2011), for goldfish during parasite infection (Wang and Belosevic 1994), for Atlantic salmon during lice infection (Krasnov et al. 2015) and for zebrafish during viral infection (López-Muñoz et al. 2015) or *Microcystis aeruginosa* infection (Liu et al. 2018). E2 probably modulates the immune response by affecting the activity of leukocytes involved in the innate response. For example, rainbow trout infected with *Yersenia ruckeri* after feeding for two weeks (short exposure) or for 5 months (long exposure) with E2 displayed downregulation of the expression of several immune-related genes in the liver such as *c3-1, c3-3* and *factor-H* (Wenger et al. 2011). Similar studies found that in *Edwardsiella ictururi* infected fish, E2 downregulated expression of the hepcidin 1 gene (Robertson et al. 2009).

Interestingly, we also found that in both seasons, infection differentially affected the level of E2. As mentioned before, we noted a high concentration of this hormone in the control fish during autumn (November/December), while it was low in the spring season (March/April). However, at 24 hpi, the level of E2 decreased in the autumn and now showed similarly low levels in both seasons. In the autumn, at 96 hpi the level of E2 increased to the level observed in control fish from this season, while it did not change in infected fish during springtime. Also, in other fish species, infection-induced changes in E2 levels were observed. For example, in Atlantic salmon, a decrease in E2 after infection with the ectoparasite salmon louse was shown at 3- and 16-days post-infection (Krasnov et al. 2015). Most probably, the changes in E2 level may have an immunomodulatory character.

In conclusion, we observed that the antibacterial/immune response of carp differed between seasons. We found season-dependent changes in the estrogen system, indicating an immunomodulatory role for this hormone. Moreover, we demonstrated that season differentially affects the estrogenic and immune activity of the head kidney and liver. These results reinforce our previous findings that the endocrine and immune systems cooperate in maintaining homeostasis and fighting infection.

### Supplementary information

ESM 1 (DOCX 78.0 kb)

## Data Availability

The data will be made available by the authors upon request. The datasets generated during and/or analyzed during the current study will be available in the https://uj.rodbuk.pl/.
