# Supplementary material for: Season affects the estrogen system and the immune response of common carp
Source: Fish Physiol Biochem. 2023 Dec 29;50(2):797–812. doi: 10.1007/s10695-023-01286-2 (PMC11021253; doi:10.1007/s10695-023-01286-2)
Supplement: Supplementary file 1 — (DOCX 78.0 kb) [file 10695_2023_1286_MOESM1_ESM.docx]

Supplementary Material

Season affects the estrogen system and the immune response of common carp

Magdalena Maciuszek^1*^, Lukasz Pijanowski^1^, Lidy Verburg-van Kemenade^2^, Magdalena Chadzińska^1^

^1^Department of Evolutionary Immunology, Institute of Zoology and Biomedical Research, Faculty of Biology, Jagiellonian University, Krakow, Poland

^2^Cell Biology and Immunology Group, Department of Animal Sciences, Wageningen University, Wageningen, the Netherlands

**Corresponding Author**
[magdalena.maciuszek@uj.edu.pl](mailto:magdalena.maciuszek@uj.edu.pl)

ORCID: <https://orcid.org/0000-0002-4733-7319>

**Figure S1. Season-dependent and infection-induced changes in the level of 17**$\boldsymbol{\beta}$**-estradiol.** Fish were kept intact (CTR) or were i.p. injected with *A. salmonicida* (4 x 10^8^ bacteria in 250 µl PBS per fish). At 24 and 96 h post-infection (hpi) blood plasma was collected. Averages and SE (n=5-7). Mean values not sharing letters are statistically different between autumn (orange bar) and spring (green bar).

**Figure S2. Level of cortisol in common carp blood plasma.** Fish were kept intact (CTR) or were i.p. injected with *A. salmonicida* (4 x 10^8^ bacteria in 250 µl PBS per fish) At 24 and 96 h post-infection (hpi) blood plasma was collected. Averages and SE (n=5-6) measured in autumn (orange bar) and spring (green bar).

**Supplementary table 1.** Primers used for quantitative RT-qPCR analysis.

| **Gene** | **Primer forward (5`-3`)** | **Primer reverse (5`-3`)** | **Acc. No** | **µM** |
| --- | --- | --- | --- | --- |
| ***40s11*** | CCGTGGGTGACATCGTTACA | TCAGGACATTGAACCTCACTGTCT | AB012087 | 1 |
| ***inos*** | AACAGGTCTGAAAGGGAATCCA | CATTATCTCTCATGTCCAGAGTCTCTTCT | AJ242906 | 1 |
| ***il-1β*** | AAGGAGGCCAGTGGCTCTGT | CCTGAAGAAGAGGAGGCTGTCA | AJ245635 | 1 |
| ***il-12p35*** | TGCTTCTCTGTCTCTGTGATGGA | CACAGCTGCAGTCGTTCTTGA | AJ480354 | 1 |
| ***ifn-γ2*** | TCTTGAGGAACCTGAGCAGAA | TGTGCAAGTCTTTCCTTTGTAG | AM168523 | 1 |
| ***arginase 1*** | TGAGGAGCTTCAGCGGATTAC | CCTATTATTCCCACGCAGTGATG | AJ871264 | 1 |
| ***arginase 2*** | GGAGACCTGGCCTTCAAGCATCT | CTGATTGGCACGTCCAACT | AJ618955 | 1 |
| ***il-10*** | CGCCAGCATAAAGAACTCGT | TGCCAAATACTGCTCGATGT | AB110780 | 1 |
| ***vitellogenin*** | ﻿TGAAATTCTTCAGACCCCCATT | CATGGCCACATTGTTTGCA | ﻿AF414432.1 | 2 |
| ***c3*** | GGTTATCAAGGGGAGTTGAGCTAT | TGCTGCTTTGGGTGGATGGGT | AB016215 | 1 |
| ***crp1*** | AGCAATGCAACATTTTTCCGTC | ACTTGCGTCAAAGCCACCCAC | JQ010977 | 2 |
| ***crp2*** | GATGCTGCAGCATTTTTCAGTC | CTCCGCATCAAAGTTGCTCAAAT | JQ010978 | 2 |
| ***erα*** | ﻿ACTGCCCACAAACTCTCACC | ﻿TGGGAACTCATAGGCTCCAT | ﻿BAF99812.1 | 1 |
| ***erβ*** | ﻿CCAGGTCCATTTGTTGGAGT | ﻿TGAGGTCTGGGGAGAAAATG | ﻿BAB91218.1 | 1 |
| ***gper1*** | ﻿CGACTCTGCTTCCTTTCACC | ﻿GATCGTCACCTCAAGCCATT | ﻿XM_019067213.1 | 1 |
| ***cyp19a*** | ﻿GGTGCCCAAGACAATGTATATGG | ﻿TTGTCCGATGGTGTCTGATGG | ﻿DQ534411.1 | 1 |
| ***cyp19b*** | ﻿ATGATGGAGCAGGTCGTCAAG | ﻿TCAACGCCATCAACGTTACC | ﻿EU375456.1 | 1 |

**Supplementary table 2.** Two way-ANOVA statistical analysis for RT-qPCR data and ELISA data, the effect of the interaction between the two factors (season and infection); the effect of infection time or the effect of season (autumn between spring). Fish were kept intact as control or injected with *A. salmonicida* (4 x 10^8^ bacteria in 250 µl PBS per fish) for 24 and 96 h post-infection in both season – spring and autumn. (MS= mean squares, DF = degrees of freedom, F= F ratio).

|  | | **Season vs infection** | | | **Infection (Time)** | | | **Season (Autumn vs Spring)** | | |
| --- | --- | --- | --- | --- | --- | --- | --- | --- | --- | --- |
|  |  | **MS** | **F (DFn, DFd)** | **P-value** | **MS** | **F (DFn, DFd)** | **P-value** | **MS** | **F (DFn, DFd)** | **P-value** |
| **Head kidney** | ***inos*** | 9254 | F (2, 25) = 31,73 | P<0,0001*** | 23816 | F (2, 25) = 81,65 | P<0,0001*** | 9505 | F (1, 25) = 32,59 | P<0,0001*** |
|  | ***il-1β*** | 1,182 | F (2, 28) = 0,7657 | P=0,4745 | 61,83 | F (2, 28) = 40,05 | P<0,0001*** | 0,2465 | F (1, 28) = 0,1597 | P=0,6925 |
|  | ***il-12p35*** | 53,04 | F (2, 29) = 4,114 | P=0,0267* | 76,15 | F (2, 29) = 5,906 | P=0,0071** | 30,06 | F (1, 29) = 2,332 | P=0,1376 |
|  | ***ifnγ2*** | 6,592 | F (2, 31) = 7,161 | P=0,0028** | 2,839 | F (2, 31) = 3,084 | P=0,0600 | 2,839 | F (2, 31) = 3,084 | P=0,0600 |
|  | ***arginase 1*** | 2,302 | F (2, 22) = 0,9405 | P=0,4056 | 25,44 | F (2, 22) = 10,39 | P=0,0007*** | 5,631 | F (1, 22) = 2,301 | P=0,1436 |
|  | ***arginase 2*** | 3,155 | F (2, 28) = 7,117 | P=0,0032** | 1,711 | F (2, 28) = 3,859 | P=0,0331* | 7,215 | F (1, 28) = 16,27 | P=0,0004** |
|  | ***il-10*** | 2,008 | F (2, 27) = 1,265 | P=0,2985 | 26,77 | F (2, 27) = 16,87 | P<0,0001*** | 7,091 | F (1, 27) = 4,467 | P=0,0439* |
|  | ***erα*** | 3,340 | F (2, 24) = 7,132 | P=0,0037** | 2,845 | F (2, 24) = 6,075 | P=0,0073** | 0,03621 | F (1, 24) = 0,07733 | P=0,7833 |
|  | ***erβ*** | 3,131 | F (2, 26) = 3,972 | P=0,0312* | 1,362 | F (2, 26) = 1,728 | P=0,1974 | 0,6687 | F (1, 26) = 0,8484 | P=0,3655 |
|  | ***gper1*** | 2,520 | F (2, 23) = 9,379 | P=0,0011** | 0,3680 | F (2, 23) = 1,370 | P=0,2742 | 0,1672 | F (1, 23) = 0,6221 | P=0,4383 |
|  | ***cyp19a*** | 18,28 | F (2, 22) = 19,62 | P<0,0001*** | 4,949 | F (2, 22) = 5,312 | P=0,0131* | 1,859 | F (1, 22) = 1,996 | P=0,1717 |
|  | ***cyp19b*** | 0,8988 | F (2, 25) = 1,539 | P=0,2342 | 7,559 | F (2, 25) = 12,95 | P=0,0001*** | 0,9071 | F (1, 25) = 1,553 | P=0,2242 |
| **Liver** | ***inos*** | 726,5 | F (2, 32) = 3,938 | P=0,0296* | 2497 | F (2, 32) = 13,53 | P<0,0001*** | 1451 | F (1, 32) = 7,864 | P=0,0085** |
|  | ***il-1β*** | 266,3 | F (2, 30) = 1,872 | P=0,1713 | 2960 | F (2, 30) = 20,81 | P<0,0001*** | 272,3 | F (1, 30) = 1,914 | P=0,1767 |
|  | ***il-12p35*** | 626,2 | F (2, 26) = 5,559 | P=0,0098** | 716,0 | F (2, 26) = 6,356 | P=0,0057** | 1685 | F (1, 26) = 14,95 | P=0,0007** |
|  | ***ifnγ2*** | 36,14 | F (2, 24) = 3,820 | P=0,0363* | 95,14 | F (2, 24) = 10,06 | P=0,0007** | 84,07 | F (1, 24) = 8,886 | P=0,0065** |
|  | ***arginase 1*** | 67,56 | F (2, 31) = 3,193 | P=0,0549 | 53,54 | F (2, 31) = 2,530 | P=0,0960 | 269,2 | F (1, 31) = 12,72 | P=0,0012** |
|  | ***arginase 2*** | 57,08 | F (2, 30) = 12,06 | P=0,0001*** | 87,93 | F (2, 30) = 18,57 | P<0,0001*** | 133,1 | F (1, 30) = 28,11 | P<0,0001*** |
|  | ***il-10*** | 16,67 | F (2, 26) = 3,393 | P=0,0490* | 12,86 | F (2, 26) = 2,617 | P=0,0921 | 70,82 | F (1, 26) = 14,41 | P=0,0008** |
|  | ***erα*** | 2,909 | F (2, 28) = 0,7380 | P=0,4871 | 23,98 | F (2, 28) = 6,084 | P=0,0064** | 8,596 | F (1, 28) = 2,181 | P=0,1509 |
|  | ***erβ*** | 0,2327 | F (2, 29) = 0,4141 | P=0,6648 | 0,7261 | F (2, 29) = 1,292 | P=0,2900 | 0,3644 | F (1, 29) = 0,6484 | P=0,4272 |
|  | ***gper1*** | 480,6 | F (2, 25) = 4,173 | P=0,0273* | 433,6 | F (2, 25) = 3,765 | P=0,0372* | 791,3 | F (1, 25) = 6,871 | P=0,0147* |
|  | ***cyp19a*** | 60,32 | F (2, 25) = 3,600 | P=0,0423* | 48,17 | F (2, 25) = 2,875 | P=0,0752 | 166,7 | F (1, 25) = 9,952 | P=0,0042** |
|  | ***cyp19b*** | 54,45 | F (2, 26) = 3,834 | P=0,0347* | 50,09 | F (2, 26) = 3,527 | P=0,0441* | 76,99 | F (1, 26) = 5,421 | P=0,0279* |
|  | ***c3*** | 0,4087 | F (2, 30) = 1,214 | P=0,3112 | 0,8526 | F (2, 30) = 2,532 | P=0,0963 | 2,018 | F (1, 30) = 5,994 | P=0,0204** |
|  | ***crp1*** | 0,1648 | F (2, 28) = 2,161 | P=0,1340 | 2,147 | F (2, 28) = 28,16 | P<0,0001*** | 0,1351 | F (1, 28) = 1,772 | P=0,1939 |
|  | ***crp2*** | 1,880 | F (2, 25) = 11,70 | P=0,0003*** | 1,758 | F (2, 25) = 10,94 | P=0,0004** | 2,892 | F (1, 25) = 17,99 | P=0,0003** |
|  | ***vtg*** | 6071872 | F (2, 29) = 5,071 | P=0,0129* | 6138660 | F (2, 29) = 5,127 | P=0,0124* | 23984651 | F (1, 29) = 20,03 | P=0,0001*** |
| **Blood** | ***estradiol*** | 9931 | F (2, 30) = 2,432 | P=0,1050 | 11855 | F (2, 30) = 2,904 | P=0,0703 | 78711 | F (1, 30) = 19,28 | P=0,0001*** |
|  | ***cortisol*** | 477,8 | F (2, 29) = 0,9460 | P=0,4000 | 1544 | F (2, 29) = 3,056 | P=0,0625 | 64,74 | F (1, 29) = 0,1282 | P=0,7229 |
